# Peer-provided Problem Management Plus (PM+) for adult Syrian refugees: a pilot randomised controlled trial on effectiveness and cost-effectiveness

**DOI:** 10.1017/S2045796020000724

**Published:** 2020-08-18

**Authors:** A. M. de Graaff, P. Cuijpers, D. McDaid, A. Park, A. Woodward, R. A. Bryant, D. C. Fuhr, B. Kieft, E. Minkenberg, M. Sijbrandij

**Affiliations:** 1 Department of Clinical, Neuro- and Developmental Psychology, Amsterdam Public Health Institute, Vrije Universiteit Amsterdam, Amsterdam, The Netherlands; 2 Department of Health Policy, Care Policy and Evaluation Centre, London School of Economics and Political Science, London, UK; 3 KIT Health, KIT Royal Tropical Institute, Amsterdam, The Netherlands; 4 School of Psychology, University of New South Wales, Sydney, NSW, Australia; 5 Department of Health Services Research and Policy, Public Health and Policy, London School of Hygiene and Tropical Medicine, London, UK; 6 i-Psy, Parnassia Groep, Almere, The Netherlands; 7 i-Psy, Parnassia Groep, Den Haag, The Netherlands

**Keywords:** Common mental disorders, lay counsellors, posttraumatic stress disorder, randomised controlled trials, task sharing

## Abstract

**Aims:**

Common mental disorders are highly prevalent among Syrian refugees. Problem Management Plus (PM+) is a brief, transdiagnostic, non-specialist helper delivered, psychological intervention targeting psychological distress. This single-blind pilot randomised controlled trial (RCT) on PM+ delivered by peer-refugees examined trial procedures in advance of a definitive RCT, evaluated PM+ 's acceptability and feasibility, and investigated its likely effectiveness and cost-effectiveness among Syrian refugees in the Netherlands.

**Methods:**

Adult Syrian refugees (*N* = 60) with elevated psychological distress (Kessler Psychological Distress Scale (K10) score >15) and reduced pychosocial functioning (WHO Disability Assessment Schedule 2.0 (WHODAS) score >16) were randomised into PM+ in addition to care as usual (CAU) (PM+/CAU; *n* = 30) or CAU alone (*n* = 30). Primary outcomes were symptoms of depression and anxiety (Hopkins Symptom Checklist; HSCL-25) at 3-month follow-up. Secondary outcomes were pychosocial functioning (WHO Disability Assessment Schedule; WHODAS 2.0), symptoms of posttraumatic stress disorder (PTSD) (PTSD Checklist for DSM 5; PCL-5) and self-identified problems (Psychological Outcomes Profiles; PSYCHLOPS). Changes in service utilisation and time out of employment and/or adult education were estimated (adapted version of the Client Service Receipt Inventory; CSRI). Semi-structured interviews on the implementation of PM+ were conducted with stakeholders (i.e. six PM+ participants, five non-specialist helpers and five key informants).

**Results:**

Recruitment, randomization and blinding procedures were successful. PM+ was generally perceived positively by stakeholders, especially regarding the intervention strategies, accommodation of the intervention and the helpers. Two serious adverse events not attributable to the trial were reported. At 3-month follow-up, the HSCL-25 total score was significantly lower for the PM+/CAU group (*n* = 30) than CAU group (*n* = 30) (*p* = 0.004; *d* = 0.58). Significant differences in favour of PM+/CAU were also found for WHODAS psychosocial functioning (*p* = 0.009, *d* = 0.73), PCL-5 symptoms of PTSD (*p* = 0.006, *d* = 0.66) and PSYCHLOPS self-identified problems (*p* = 0.005, *d* = 0.81). There were no significant differences in mean health service costs (*p* = 0.191) and the mean costs of lost productive time (*p* = 0.141). This suggests PM+ may potentially be cost-effective with an incremental cost from a health system perspective of €5047 (95% CI €0–€19 773) per additional recovery achieved.

**Conclusions:**

Trial procedures and PM+ delivered by non-specialist peer-refugee helpers seemed acceptable, feasible and safe. Analyses indicate that PM+ may be effective in improving mental health outcomes and psychosocial functioning, and potentially cost-effective. These results support the development of a definitive RCT with a larger sample of refugees and a longer follow-up period.

## Introduction

Recent years have witnessed a dramatic increase in the number of asylum seekers and refugees worldwide, largely accounted for by refugees fleeing from the war in Syria to its neighbouring countries and Europe (UNHCR, 2018). Common mental disorders are prevalent in refugees, with estimated prevalence rates of 30.8% for depression and 30.6% for posttraumatic stress disorder (PTSD) (Steel *et al*., 2009). Recent studies among Syrian refugees specifically also report elevated levels of distress (Tinghög *et al*., 2017; Poole *et al*., 2018).

Refugees are individuals who are ‘unable or unwilling to return to their country of origin owing to a well-founded fear of being persecuted’ (UN General Assembly, 1951). The steep increase in refugees carries significant public health implications (Priebe *et al*., 2016). Since the outbreak of the Syrian war, there has been an increase in studies evaluating mental health and psychosocial support programmes for Syrian refugees in Europe (e.g. Lehnung *et al.*, 2017) and the Middle East (e.g. Weinstein *et al.*, 2016). Meta-analytic evidence supports cognitive behavioural therapy (CBT) and narrative exposure therapy to treat PTSD in refugees (Nosè *et al*., 2017; Turrini *et al*., 2017). Although high-income countries such as the Netherlands have specialised mental health staff and programmes available for refugees, the treatment gap is large. Studies among refugees/migrants found that a large proportion did not receive adequate mental health care (Lamkaddem *et al*., 2014; Priebe *et al*., 2016). Access to specialist mental health care is hampered by various barriers such as long waitlists, communication difficulties and stigma (Satinsky *et al*., 2019).

To overcome barriers to mental health care for communities affected by adversity, the World Health Organization (WHO) developed Problem Management Plus (PM+). PM+ is a brief, transdiagnostic psychological intervention targeting symptoms of depression, anxiety and distress, and is based on CBT and problem-solving therapy strategies (Dawson *et al*., 2015). The intervention comprises five face-to-face sessions with a non-specialist helper (Dawson *et al*., 2015). Randomised controlled trials (RCTs) on individual PM+ in violence-affected communities in Pakistan and Kenya showed that participants who received individual PM+ had fewer symptoms of depression, anxiety and PTSD, higher levels of functioning and fewer self-identified problems (Rahman *et al*., 2016; Bryant *et al*., 2017). Another RCT on group PM+ in distressed females in the Swat area in Pakistan showed similar results (Rahman *et al*., 2019).

In this study, we conducted a pilot RCT to (1) test trial procedures in advance of the definitive RCT; (2) evaluate acceptability and feasibility; and (3) gain a preliminary understanding about the likely effectiveness and cost-effectiveness of PM+ among Syrian refugees with elevated levels of psychological distress in the Netherlands.

## Methods

### Setting

The study was carried out by the Vrije Universiteit Amsterdam (VU) in collaboration with Stichting Nieuw Thuis Rotterdam (SNTR), a non-governmental organization (NGO) providing support with integration, including housing, Dutch language courses and guidance to work to approximately 200 Syrian refugee families with resident status in Rotterdam. The trial was approved by the Research Ethics Review Committee at VU Medical Center, the Netherlands (Protocol ID: NL61361.029.17, 7 September 2017) and prospectively registered online (https://www.trialregister.nl/trial/6665).

### Study design and participants

A single-blind pilot RCT using mixed-methods was conducted from April 2018 to May 2019. The study is part of the larger EU H2020-funded STRENGTHS project, which aims to scale-up brief, psychological interventions among Syrian refugees in Europe and the Middle East (Sijbrandij *et al*., 2017). CONSORT and CHEERS reporting checklists (Husereau *et al*., 2013; Eldridge *et al*., 2016) are appended (Checklist A1 and A2).

Adult Arabic-speaking Syrian refugees (18 years or above) were recruited during language classes and individual home visits by SNTR staff. Inclusion criteria were elevated levels of psychological distress as indicated by a score >15 on the Kessler Psychological Distress Scale (K10) (Kessler *et al*., 2002) and impaired daily functioning, indicated by a score >16 on the WHO Disability Assessment Schedule 2.0 (WHODAS 2.0; Ustun *et al*., 2010). Exclusion criteria were acute medical conditions, imminent suicide risk (assessed with the PM+ manual suicidal thoughts interview) (WHO, 2016), expressed acute needs or protection risks, indications of severe mental disorders (e.g. psychotic disorders) or cognitive impairment (e.g. severe intellectual disability; assessed by the PM+ manual observation checklist) (WHO, 2016).

### Procedures

Oral and written informed consent (IC) was obtained from all participants before screening. Included participants completed baseline assessment questionnaires and were randomised into PM+/care as usual (CAU) or CAU alone by an independent researcher not involved in the study. CAU comprises all other mental health services available to Syrian refugees in the Netherlands (e.g. community services and non-directive counselling by local NGOs, or referral to specialised PTSD treatment such as narrative exposure therapy). Randomization with block size 6 was performed using software on a 1:1 basis. Participants were phoned on group allocation by an Arabic-speaking team member not involved in assessments. Participants randomised to PM+/CAU were directly contacted by their helper to plan the first session within 1 week after baseline assessment.

Post- and 3-month follow-up assessments were scheduled 1 week after the 5th PM+ session (or 6 weeks after baseline) and 3 months after the 5th PM+ session. Assessments were carried out by two Arabic-speaking assessors who received a 3-day training on questionnaire administration, general interview techniques, common mental disorders, psychological first aid and ethical research conduct. Assessors were blinded to condition and indicated after each post- and follow-up assessment whether group allocation was disclosed to them (i.e. yes PM+/CAU; yes CAU; no).

### Problem Management Plus (PM+)

The PM+ intervention has five 90 min sessions, delivered weekly. Four components are introduced by the helper, including a slow breathing exercise, problem-solving strategy, behavioural activation through re-engaging with pleasant and task-oriented activities, and accessing social support. A detailed description of PM+ is available (Dawson *et al*., 2015). The PM+ manual was translated/culturally adapted for use among Syrian refugees through qualitative study (cf. Applied Mental Health Research Group, 2013) and by using a framework for the cultural adaptation of psychological interventions (Bernal and Sáez-Santiago, 2006; see de Graaff *et al*., 2020).

The intervention was delivered by eight Arabic-speaking Syrian non-specialist helpers already working as ‘connectors’ at SNTR. They received 8 days of training followed by weekly face-to-face group supervision by PM+ trainers/supervisors throughout the trial. Training involved education about common mental disorders, basic counselling skills, delivery of intervention strategies and self-care (WHO, 2016). Supervision included discussion of individual cases and difficulties experienced by helpers, practice of skills and self-care (WHO, 2016). Helpers had at least high school education, a background in social work, teaching or another related field, and sufficient Dutch or English speaking ability. Trainers/supervisors were mental health care professionals who underwent 5-day training covering elements of the training of helpers, as well as training and supervision skills (cf. Rahman *et al*., 2016).

A 25% random sample of the audio recordings was independently coded by two research assistants for adequate delivery of PM+ treatment elements (yes/no) through a checklist addressing requisite PM+ components per session (see appended Checklist A3).

### Primary outcome measure

*Symptoms of anxiety and depression.* The 25-item Hopkins Symptom Checklist (HSCL-25) (Arabic version) (Derogatis *et al*., 1974; Selmo *et al*., 2016) was used to measure symptoms of anxiety (10 items) and depression (15 items). Item mean scores (range 1–4) were analysed.

### Secondary outcome measures

*Functional impairment.* The WHODAS 2.0 is a well-accepted, validated 12-item instrument to assess health and disability (Ustun *et al.*, 2010). Items are rated on a 1–5 scale (range 12–60).

*Posttraumatic stress symptoms.* The Arabic version of the 20-item PTSD Checklist for DSM-5 (PCL-5) (Blevins *et al*., 2015; Ibrahim *et al*., 2018) assesses PTSD symptoms on a 0–4 scale (range 0–80).

*Self-identified problems.* The Psychological Outcomes Profiles (PSYCHLOPS) (Ashworth *et al*., 2004) questionnaire is a patient-generated indicator of change after therapy. It covers self-identified problems and function (both free-text fields), and wellbeing scored on a 0–5 scale (range 0–20).

*Client Service Receipt Inventory (CSRI)*. The CSRI (Beecham and Knapp, 1992) was modified for use in Syrian refugees in the Netherlands to self-report health service utilization, receipt of informal family care and participation in employment/education or other productive use of time over 3 months.

### Other measures

*Trauma exposure.* Life-time traumatic experiences were measured through a 27-item checklist (Schick *et al*., 2016) adapted for this project. Items were scored 1 (yes) or 0 (no), total range 0–27.

*Post-migration stressors*. The Post-Migration Living Difficulties checklist (Arabic version) (Silove *et al*., 1997; Schick *et al*., 2016) assesses 17 post-migration challenges scored on a 0–4 scale. Items with at least a score of 2 (moderately serious problem) were considered positive responses and summed for analysis (range 0–17).

These measures were used for descriptive analyses.

### Quantitative and qualitative analyses

To compare reductions in primary/secondary outcomes between groups across three time points in the intention-to-treat sample (*N* = 60), we used linear mixed models in RStudio version 3.6.1 (R Core Team, 2018). This method allows the number of observations to vary between participants and handles missing outcome data. The mixed model uses a longitudinal data structure that includes both fixed and random effects. Time (categorical), group (PM+/CAU *v*. CAU) and interactions between group and time were included as fixed effects in mixed models together with a random intercept and random time effect. Differences in least-squares means (intervention effects) at each time point with 95% confidence intervals were derived. Cohen's *d* for the effect of the intervention was estimated by calculating the difference between estimated means (corrected for baseline) divided by raw pooled standard deviation. A two-sided *p* < 0.05 indicated statistical significance. As this study's primary intention was to test feasibility and acceptability, it was not powered to detect significant differences.

The reliable change index was used to evaluate whether participants have reliable and clinically significant change scores from baseline to post- and follow-up (Jacobson and Truax, 1991).

The economic analysis was performed from both a healthcare system and a broader perspective, including productivity impacts on participants and their families. Health service utilization and productivity losses were estimated across all time points. PM+ training, supervision resource and delivery costs were obtained from project records. Unit costs were attached to health service utilization using published tariffs used in the Netherlands (Hakkaart-van Roijen *et al*., 2015). Medication reimbursement rates were obtained from the Netherlands National Health Care Institute (Zorginstituut Nederland, 2020). See Appendix Table A1 for unit costs used. All patient and family productivity losses were valued using age-specific 2018 minimum wage rates (Government of the Netherlands, 2020). All costs are 2018 euros and discounting was not applied given the short study duration. Given the skewed distribution of costs, differences in mean costs were compared between the two groups using bias-corrected and accelerated (BCa) bootstrapping 1000 times. Incremental cost-effectiveness ratios (ICERs) per additional recovery and improvement without recovery at 3-month follow-up were calculated. Statistical uncertainty was explored through bootstrapping 1000 randomly resampled pairs of costs and outcomes. Cost-effectiveness acceptability curves (CEACs) were generated to show the likelihood PM+ is cost-effective at different willingness to pay levels.

Semi-structured interviews were audio recorded and transcribed verbatim in interview language (Arabic, Dutch or English). Arabic transcripts were translated into English by bi-lingual research assistants. Deidentified transcripts were analysed thematically and independently by two researchers (AdG and AW) in NVivo (QSR International Pty Ltd, 2015). Inductive analysis was used to categorise data and elicit themes. Findings were discussed by these researchers and a final coding framework agreed and then applied to all transcripts.

## Results

### Objective 1: testing trial procedures

#### Recruitment and consent rates

**Fig. 1.**
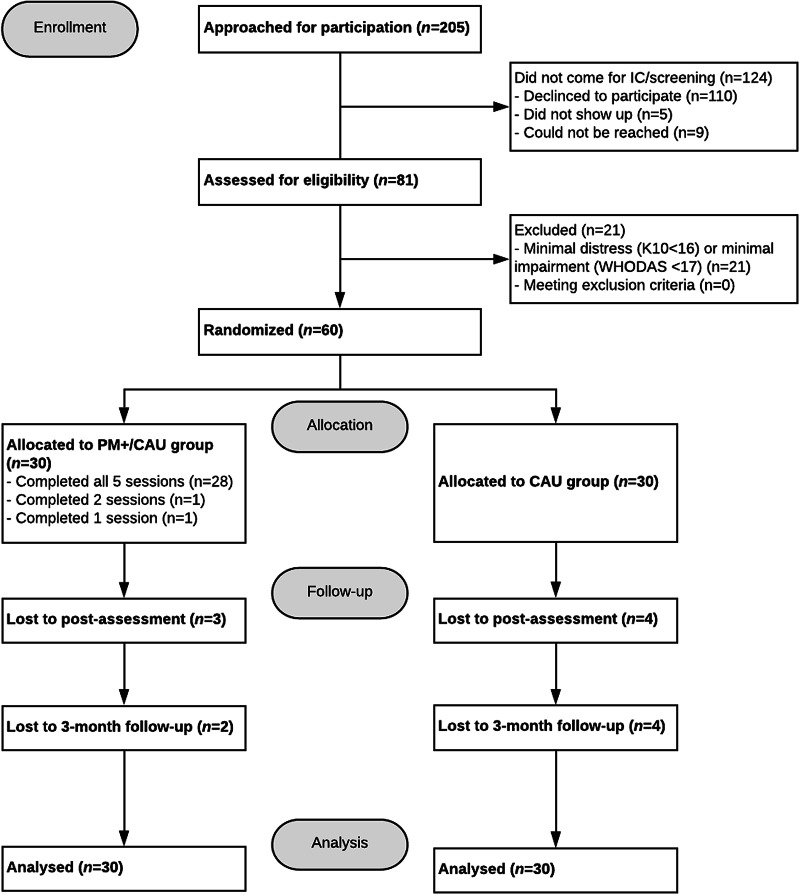
CONSORT flow-diagram.

Recruitment occurred from April to November 2018. In total, 205 individuals gave permission to SNTR staff to be contacted by the VU research team. Of these, 110 declined, ten were unreachable and five did not attend screening. Eighty-one participants gave IC and were screened for eligibility to participate. Of these, 60 participants were included and randomised into PM+/CAU (*n* = 30) or CAU alone groups (*n* = 30). Figure 1 presents the CONSORT flow diagram.

#### Group allocation and blinding

An SNTR team-leader matched PM+ participants to helpers. During the trial, we decided to ask all participants at the screening interview whether they preferred matching with a male or female helper to facilitate the matching procedures; however, female helpers preferred not to be matched to male participants. Blinding was successful in 47% of outcome assessments.

#### Attendance and follow-up assessments

Assessments occurred between May 2018 and March 2019. Twenty-eight (93.3%) of the 30 participants allocated to PM+/CAU completed all five sessions. Two stopped after sessions 1 and 2 because the spouse did not allow participation and due to lack of time. Three months post-intervention, data were obtained from 54 participants, with 86.7% completing all assessments. Reported reasons for non-attendance across assessments were ‘prefers to withdraw’ (*n* = 3), ‘lack of time’ (*n* = 3), ‘abroad/unavailable’ (*n* = 1) and ‘no approval from spouse’ (*n* = 1). In 23% of outcome assessments, assessors assisted participants by reading questionnaires aloud.

#### PM+ protocol fidelity assessment

Fifty per cent (*n* = 15) of PM+/CAU participants agreed to have their sessions audiorecorded. Two research assistants scored 25% (*n* = 18 tapes) of 72 available audiorecorded sessions after five practice tapes scored by both research assistants (Cohen's *κ* = .80). Fidelity checks indicated helpers adhered to 76.6% of the PM+ protocol. Helpers completed 27 checklists, reporting 93.7% completion of the PM+ protocol.

### Objective 2: experiences with PM+ and barriers to treatment

Semi-structured interviews with six PM+ participants, five helpers and five key informants explored experiences with PM+, acceptability and feasibility in addition to barriers and facilitators of PM+ implementation. Quotes (Q) are depicted in Appendix Table A2.

#### Experiences with the PM+ intervention

Interviewees spoke generally positively about the PM+ intervention (Q1.1) and strategies (Q1.2–3). Its therapeutic effect on participant mental health (Q1.5–6), as well as that of helpers (Q1.7), was the dominant reason for holding a positive view.

Helpers stressed breaking homework into small feasible steps would improve adherence (Q1.8). According to helpers, participants generally became more motivated and confident about the intervention after their first positive experience in applying a learned strategy. Some participants said they started to forget about strategies after completing PM+ (Q1.9), and several participants and helpers thought PM+ might be too brief (Q1.10–12).

Perceived facilitators of PM+ adherence were related to the accommodation of the programme for participants (e.g. travel expense coverage, flexibility scheduling sessions) and helpers (e.g. time reserved to conduct PM+ sessions and supervision during working hours, ability to decrease caseload) (Q1.13–15).

Overall, participants did not disclose their participation in PM+ for fear of gossip and ridicule (Q1.16). However, a few said they shared their PM+ experiences with family members and practised strategies with them. A challenge to intervention adherence was the ‘busy lives’ (H1) of some participants.

#### Views on the helper

Participants and helpers mentioned they quickly established rapport and trust and felt comfortable sharing their stories and problems (Q2.1). Two participants even called their helper a ‘friend’ (Q2.2).

All interviewees spoke about the importance of PM+ being in Arabic, as it facilitated communication and self-expression (Q2.3–4). Having a similar background was generally found supportive for building rapport, although it was mentioned that it could cause distrust. For example, one participant was initially apprehensive because the helper was Syrian but later found their shared experiences beneficial (Q2.5). Participants appreciated similarities with their helper, but also that the helper was otherwise a stranger (Q2.6–8), creating a confidential and safe environment (Q2.9).

Generally, participants perceived their helper as competent (Q2.10–11). One participant commented that if the helper was a professional, he may have benefitted more (Q2.12).

Information on experiences in training and supervision of helpers is provided in Appendix Table A2.

### Objective 3: estimating likely effectiveness and cost-effectiveness

#### Treatment effect

Two serious adverse events related to domestic violence were reported to the Research Ethics Review Committee. Table 1 shows demographic characteristics, traumatic events and post-migration stressors. Table 2 presents findings on primary outcomes of anxiety and depression (HSCL-25), and secondary outcomes of functional impairment (WHODAS 2.0), symptoms of PTSD (PCL-5) and self-identified problems (PSYCHLOPS) in PM+/CAU and CAU groups at all time points. There were no significant group differences between participants who were lost to follow-up *v*. those retained. The pattern of missing data is presented in Appendix Table A3.

**Table 1.** Demographic characteristics

	Total sample (*N* = 60)	PM + /CAU (*n* = 30)	CAU (*n* = 30)
Age, M (s.d.)	38.1 (12.2)	37.6 (11.8)	38.6 (12.7)
Sex, male *n* (%)	24 (40.0)	12 (40.0)	12 (40.0)
Marital status *n* (%),^a^ Never married	6 (10.2)	3 (10.3)	3 (10.0)
Currently married	42 (71.2)	20 (69.0)	22 (73.3)
Separated	4 (6.8)	2 (6.7)	2 (6.7)
Divorced	6 (10.2)	3 (10.0)	3 (10.0)
Widowed	1 (1.7)	1 (3.3)	0 (0.0)
Cohabiting	0 (0.0)	0 (0.0)	0 (0.0)
Education (highest started), *n* (%)^a^			
No or basic education	30 (50.8)	15 (51.7)	15 (50.0)
Secondary education	20 (33.9)	7 (24.1)	13 (43.4)
Higher education (at least Bachelor)	9 (15.3)	7 (24.1)	2 (6.7)
Post-migration stressors, M (s.d.)	7.0 (3.3)	7.3 (3.6)	6.6 (3.0)
Total number of life-time traumatic events, M (s.d.)	10.2 (6.2)	9.0 (5.7)	11.5 (6.5)
Most reported traumatic event types, *n* (%), Civilian in war zone	46 (76.7)	23 (76.7)	23 (76.7)
In danger during the flight	40 (66.7)	21 (70.0)	19 (63.3)
Forced separation from family member	37 (61.7)	17 (56.7)	20 (66.7)
Unnatural death of family member or friend	37 (61.7)	18 (60.0)	19 (63.3)
Lack of shelter	35 (58.3)	17 (56.7)	18 (60.0)

a Data not obtained for one PM+/CAU participant, valid per cent reported.

**Table 2.** Summary statistics and results from mixed-model analysis of primary and secondary outcomes

		Descriptive statistics, M (s.d.)		Mixed-model analysis^a^		
Outcomes	Time point	PM+/CAU (*n* = 30)	CAU (*n* = 30)	Difference in LS mean (95% CI)	*p*-value	Effect size^a^
HSCL-25 total	Baseline	2.42 (0.54)	2.55 (0.65)			
	Post-assessment	1.86 (0.58)	2.38 (0.65)	0.45 (0.29–0.61)	0.005	0.54
	Follow-up	1.92 (0.62)	2.42 (0.59)	0.48 (0.32–0.65)	0.004	0.58
HSCL anxiety	Baseline	2.32 (0.63)	2.41 (0.63)			
	Post-assessment	1.76 (0.62)	2.22 (0.71)	0.41 (0.24–0.57)	0.017	0.48
	Follow-up	1.94 (0.68)	2.38 (0.64)	0.43 (0.25–0.60)	0.016	0.48
HSCL depression	Baseline	2.48 (0.55)	2.64 (0.72)			
	Post-assessment	1.93 (0.66)	2.48 (0.68)	0.49 (0.32–0.66)	0.005	0.50
	Follow-up	1.91 (0.63)	2.45 (0.63)	0.52 (0.35–0.70)	0.003	0.52
WHODAS 2.0	Baseline	32.13 (7.57)	30.52 (7.51)			
	Post-assessment	24.19 (9.16)	27.77 (9.37)	3.32 (1.15–5.49)	0.130	0.53
	Follow-up	23.65 (6.40)	30.15 (10.05)	6.42 (4.01–8.83)	0.009	0.73
PCL-5	Baseline	35.46 (18.04)	37.25 (17.11)			
	Post-assessment	21.41 (16.06)	34.50 (15.47)	11.13 (6.73–15.53)	0.013	0.59
	Follow-up	20.21 (17.51)	34.12 (17.14)	13.35 (8.67–18.03)	0.006	0.66
PSYCHLOPS	Baseline	15.54 (2.56)	15.47 (3.95)			
	Post-assessment	9.81 (5.92)	13.52 (4.89)	3.23 (1.83–4.63)	0.022	0.62
	Follow-up	9.71 (5.71)	15.12 (7.40)	5.25 (3.45–7.05)	0.005	0.81

M, mean; s.d., standard deviation; LS mean, least-squares mean.

HSCL-25 = 25-item Hopkins Symptoms Checklist (range item-mean = 1–4, higher scores indicate elevated anxiety or depression); WHODAS 2.0 = WHO Disability Assessment Schedule 2.0 (range 12–60, higher scores indicate worse functional impairment); PCL-5 = PTSD Checklist for DSM-5 (range 0–80, higher scores indicate greater severity); PSYCHLOPS = Psychological Outcomes Profiles (range 0–20, higher scores indicate poorer outcome).

a Effect sizes are determined by calculating the difference between the estimated means (corrected for baseline) divided by the raw pooled standard deviation.

#### Primary outcomes

**Fig. 2.**
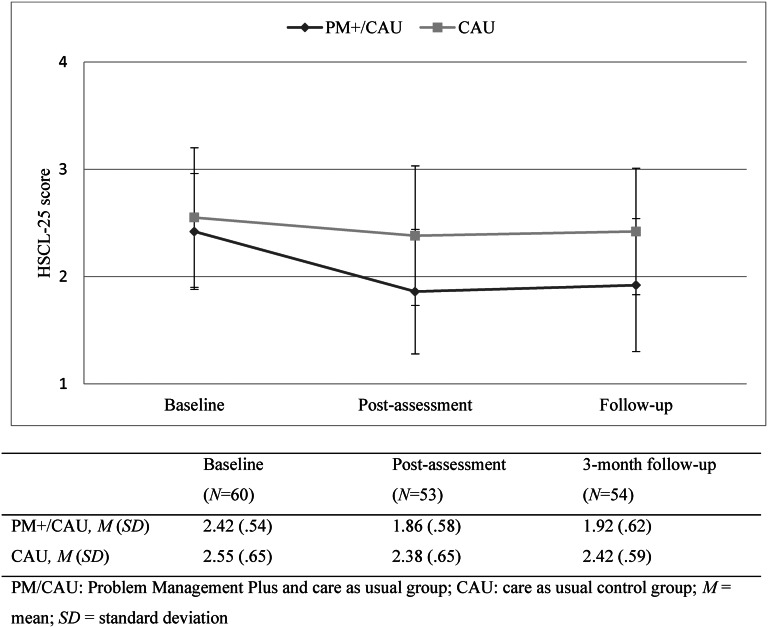
HSCL-25 total score across time points.

Linear mixed models in the intention-to-treat sample for the HSCL-25 total score showed a significant effect of time, moderated by condition (*χ*^2^(2) = 10.41; *p* = 0.005). In the PM+/CAU group, overall HSCL-25 scores decreased relative to the CAU group (see Fig. 2). Post-hoc tests showed PM+/CAU relative to the CAU had lower scores 1 week (mean [standard deviation] 1.86 [0.58] *v*. 2.38 [0.65]; adjusted mean difference (AMD), 0.45; 95% CI 0.29–0.61, *p* = 0.005) and 3 months after the intervention (1.92 [0.62] *v*. 2.42 [0.59]; AMD 0.48; 95% CI 0.32–0.65, *p* = 0.004). Effects sizes were moderate to large (*d* = 0.54 and *d* = 0.58, respectively) (Table 2).

For HSCL-25 anxiety, the effect of time was significant, and this was moderated by condition (*χ*^2^(2) = 7.58; *p* = 0.022). In the PM+/CAU group, anxiety decreased relative to the CAU group. Post-hoc contrasts showed a medium effect post-assessment (1.76 [0.62] *v*. 2.22 [0.71]; AMD 0.41; 95% CI 0.24–0.57, *p* = 0.017, *d* = 0.48), and at 3-month follow-up (1.94 [0.68] *v*. 2.38 [0.64]; AMD 0.43; 95% CI 0.25–0.60, *p* = 0.016, *d* = 0.48).

We also found a significant effect of time for HSCL-25 depression, moderated by condition (*χ*^2^(2) = 8.58; *p* = 0.013). In the PM+/CAU group, depression decreased relative to the CAU group. Post-hoc contrasts showed a medium effect post-assessment (1.93 [0.66] *v*. 2.48 [0.68]; AMD 0.49; 95% CI 0.32–0.66, *p* = 0.005, *d* = 0.50) and at 3-month follow-up (1.91 [0.63] *v*. 2.45 [0.63]; AMD 0.52; 95% CI 0.35–0.70, *p* = 0.003, *d* = 0.52).

#### Secondary outcomes

Linear mixed models furthermore showed a significant interaction effect in favour of PM+/CAU between time and condition for psychosocial functioning (WHODAS 2.0; *χ*^2^(2) = 12.99; *p* = 0.001), symptoms of PTSD (PCL-5; *χ*^2^(2) = 9.07; *p* = 0.010) and self-identified problems (PSYCHLOPS; *χ*^2^(2) = 10.51; *p* = 0.005). Post-hoc contrasts at 3-month follow-up showed the PM+/CAU group relative to the CAU group had higher levels of psychosocial functioning (23.65 [6.40] *v*. 30.15 [10.05]; AMD 6.42; 95% CI 4.01–8.83, *p* = 0.009), and decreased scores for PTSD symptoms (20.21 [17.51] *v*. 34.12 [17.14]; AMD 13.35; 95% CI 8.67–18.03, *p* = 0.006) and self-identified problems (9.71 [5.71] *v*. 15.12 [7.40]; AMD 5.25; 95% CI 3.45–7.05, *p* = 0.005). Moderate to large effects were found (*d* = 0.73, *d* = 0.66, *d* = 0.81, respectively).

Analyses with PM+ completers only (*n* = 28) *v*. CAU (*n* = 30) indicated similar results (Appendix Table A4).

#### Reliable change index for symptoms of anxiety and depression

At 3-month follow-up, 14 PM+/CAU participants had a reliable change on HSCL-25 total score, of which three were clinically significant (i.e. recovered). In the CAU group, six participants had a reliable decrease in HSCL-25 scores, while two had a reliable increase in scores (i.e. deteriorated) (Appendix Table A5).

#### Economic analysis

At 3-month follow-up, mean costs per PM+/CAU participant were significantly higher than in CAU participants from a health service perspective (€888.75 [s.d. €432.24] *v*. €347.97 [326.93]; MD €540.78; 95% CI €336.17–€755.78, *p* = 0.001) (Table 3). Excluding PM+ training, supervision and delivery, costs remained non-significantly higher for PM+/CAU (€485.65 [€432.24] *v*. €347.97 [326.93]; MD €137.67; 95% CI €–67.71 to €355.53, *p* = 0.195).

**Table 3.** Mean health and productivity costs (2018 euros) per participant at 3-month follow-up

Type of cost	PM+/CAU (*n* = 28)	CAU (*n* = 26)	Mean difference (BCa 95% CI)	*p*
PM+ intervention costs				
PM+ intervention, M (s.d.)	403.10 (0)	0 (0)	403.10 (403.10–403.10)	0.000
Health service utilisation, M (s.d.)				
Community health worker	5.14 (12.83)	2.08 (5.86)	3.07 (−1.64 to 8.63)	0.260
Community-based doctor	61.93 (79.63)	51.00 (90.85)	10.93 (−38.36 to 56.03)	0.641
Psychiatrist	45.50 (129.25)	18.85 (55.57)	26.65 (−15.59 to 86.93)	0.325
Psychologist	35.89 (155.25)	10.31 (41.05)	25.59 (−16.75 to 91.12)	0.407
Psychiatric nurse	0.00 (0.00)	3.46 (12.49)	−3.46 (−9.00 to 1.24)	0.135
Social worker	99.57 (192.76)	94.15 (150.31)	5.42 (−83.66 to 96.26)	0.908
Physiotherapist	78.93 (205.27)	40.54 (125.76)	38.39 (−44.74 to 138.50)	0.408
Other health professionals	90.64 (58.27)	96.23 (58.92)	−5.59 (−36.95 to 27.78)	0.728
Hospital inpatient stays – general health	35.36 (187.09)	0.00 (0.00)	35.36 (−29.12 to 134.97)	0.139
Hospital outpatient services – general health	30.54 (58.12)	25.58 (78.60)	4.96 (−36.28 to 41.64)	0.795
Medications	2.15 (8.33)	5.78 (15.81)	−3.63 (−11.29 to 3.23)	0.303
Total health service utilisation costs	485.65 (432.24)	347.97 (326.93)	137.67 (−67.71 to 355.53)	0.195
Total health system costs, M (s.d.)	888.75 (432.24)	347.97 (326.93)	540.78 (336.17–755.78)	0.001
Productivity losses, M (s.d.)	28.91 (102.78)	297.15 (894.29)	−268.24 (−719.91 to 6.76)	0.325
Total health and productivity costs, M (s.d.)	917.65 (453.28)	645.12 (1149.46)	272.53 (−338.81 to 711.02)	0.368

Productivity costs were non-significantly lower for PM+/CAU participants (€28.91 [102.78] *v*. €297.15 [894.29]; MD €268.24; 95% CI €–719.91 to €6.76, *p* = 0.325). Overall costs between PM+/CAU and CAU did not differ significantly (€917.65 [€453.28] *v*. €645.12 [1149.46]; MD €272.53; 95% CI €–338.81 to €711.02, *p* = 0.368).

**Fig. A1:**
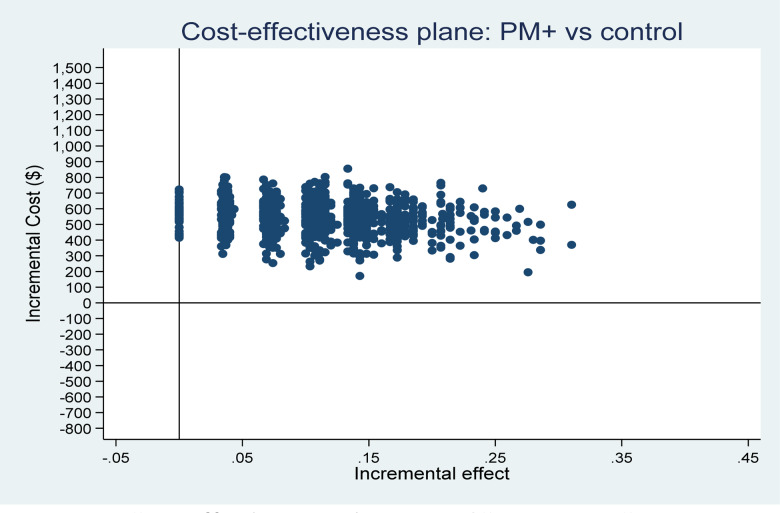
Cost-effectiveness plane: PM+/CAU *v*. CAU per recovery achieved (health system perspective).

**Fig. A2:**
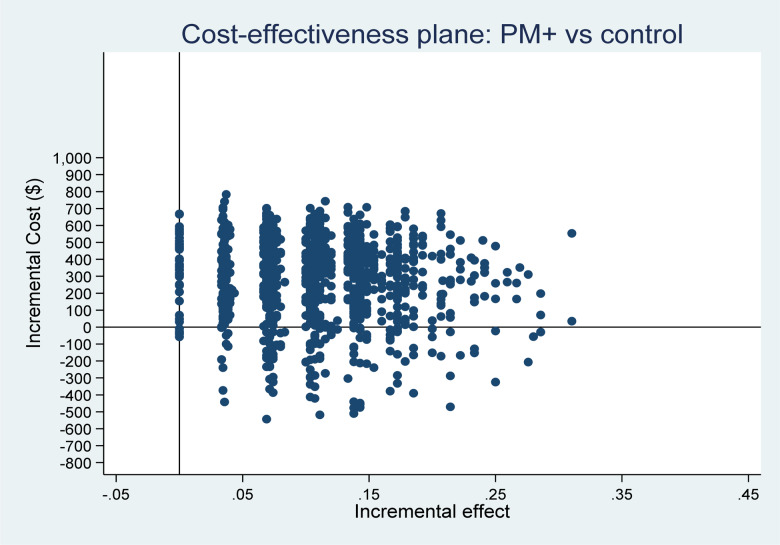
Cost-effectiveness plane: PM+/CAU *v*. CAU per recovery achieved (health system and productivity perspective).

**Fig. A3:**
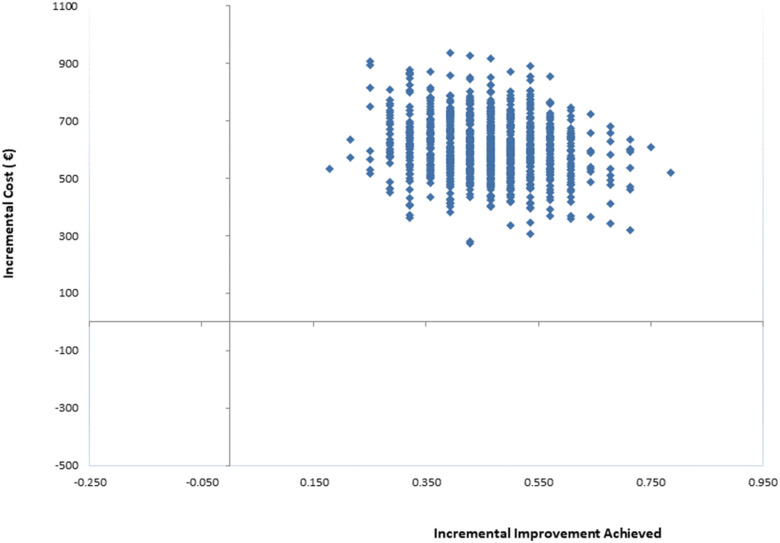
Cost-effectiveness plane: PM+/CAU *v*. CAU per improvement achieved (health system perspective).

**Fig. A4:**
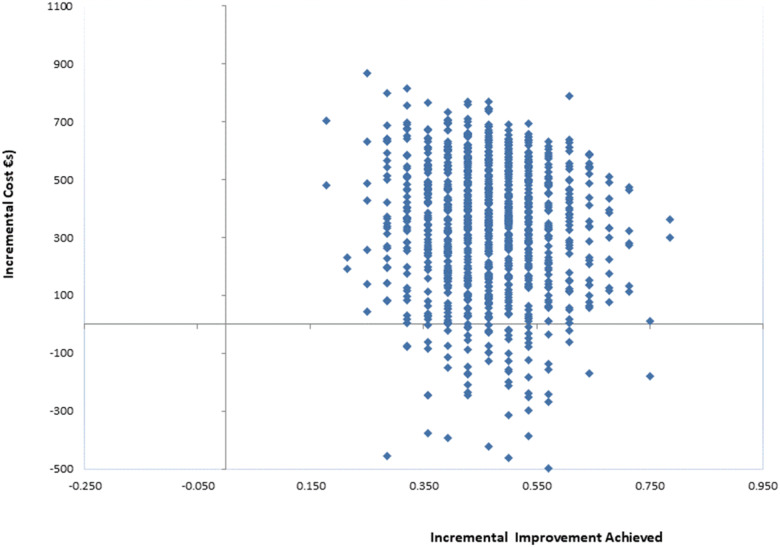
Cost-effectiveness plane: PM+/CAU *v*. CAU per recovery achieved (health system and productivity loss perspective).

**Fig. A5:**
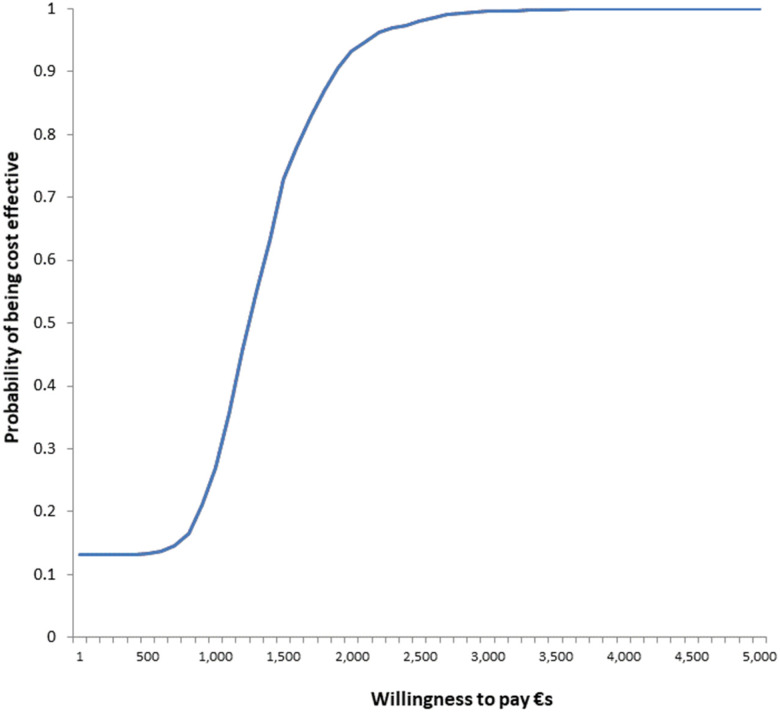
Cost-effectiveness acceptability curve: willingness to pay per improvement achieved from PM+/CAU intervention (health system perspective).

**Fig. A6:**
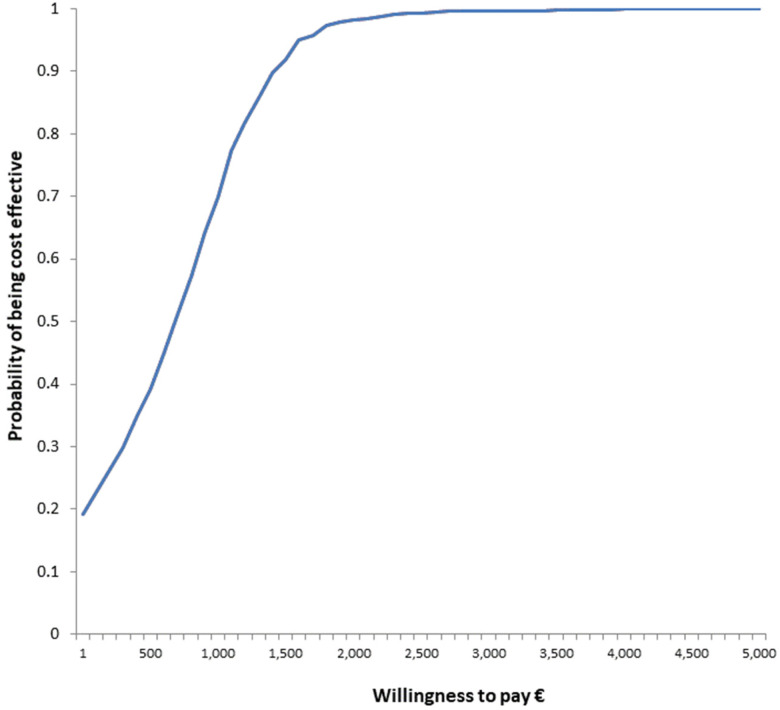
Cost-effectiveness acceptability curve: willingness to pay per improvement achieved from PM+/CAU intervention (health system perspective).

Table 4 summarises results of the exploratory cost-effectiveness analysis. From a health system perspective, PM+/CAU had an ICER of €5047 (95% CI €0–€19 773) per additional recovery achieved. This was €2266 (95% CI €–1070 to €15 930) when productivity losses averted were included. Cost-effectiveness planes in Figs A1-A2 (Appendix) indicate when productivity losses are considered PM+/CAU may even have both better outcomes and lower costs than CAU. The ICER per additional improvement on the HSCL-25 without recovery was €2009 (95% CI €0–€2406). Figs A3–A6 (Appendix) show cost-effectiveness planes and CEACs per additional improvement without recovery.

**Table 4.** Exploratory cost-effectiveness analyses (2018 euros)

	Health system perspective	Likelihood cost-effective	Health system and productivity loss perspective	Likelihood cost-effective
Incremental cost per recovery achieved at 3-month follow-up (95% CI)^a^	€5047 (€0–€19 773)	85%	€2266 (€–1070 to €15 930)	93%
Incremental cost per improvement achieved at 3-month follow-up (95% CI)^b^	€2009 (€0–€2406)	93%	€902 (€–276 to €1813)	98%

a Assumes a willingness to pay of €10 000 per recovery on the HSCL-25 achieved.

b Assumes a willingness to pay of €2000 per significant improvement on the HSCL-25 achieved.

**Fig. A7:**
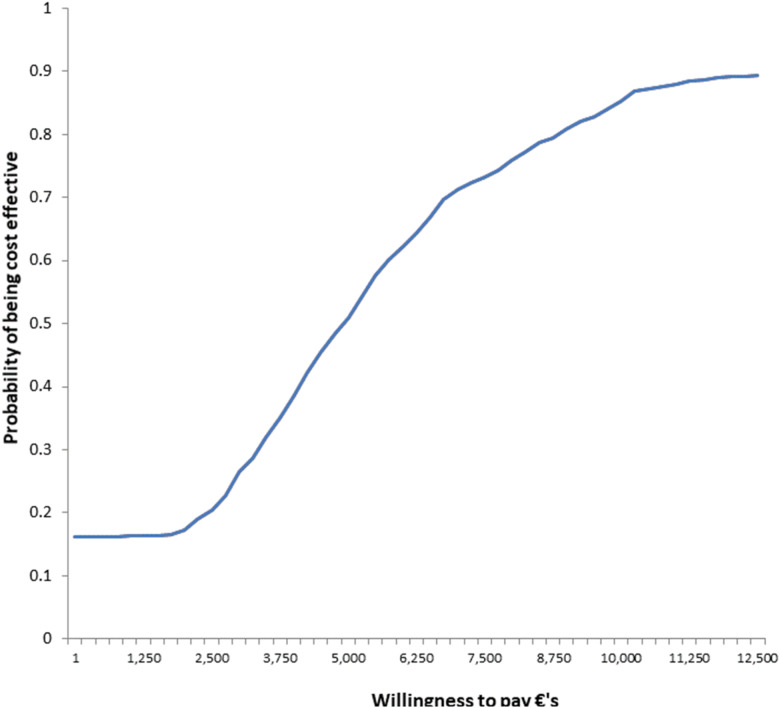
Cost-effectiveness acceptability curve: willingness to pay per recovery achieved from PM+/CAU intervention (health system perspective).

**Fig. A8:**
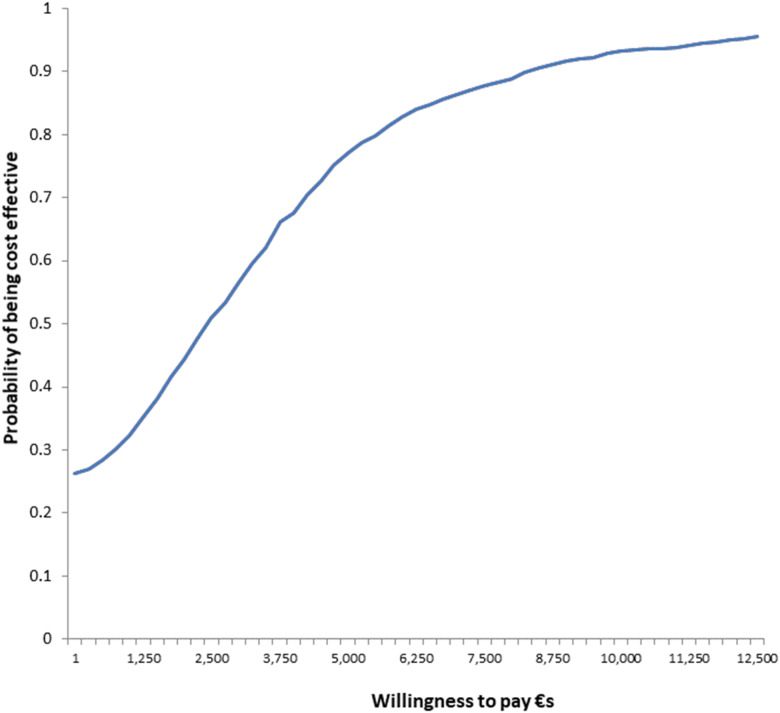
Cost-effectiveness acceptability curve: willingness to pay per recovery achieved from PM+/CAU intervention (health system and productivity perspective).

While no accepted cost-effectiveness threshold for recovery from depression and anxiety exists, CEAC indicate at least an 85% chance that PM+/CAU is cost-effective if funders are willing to pay €10 000 per recovery (Figs A7-A8 in Appendix). (In the Netherlands, €20 000 per additional year lived in full quality health is usually considered cost-effective; Brouwer *et al*. (2019).)

## Discussion

This pilot RCT aimed to test trial procedures, treatment facilitators and barriers, and likely effectiveness and cost-effectiveness of PM+ among Syrian refugees in the Netherlands. Our findings suggest that the adapted PM+ protocol is acceptable for use among Syrian refugees. Participants, helpers and key informants were generally positive about the intervention, including the PM+ strategies, accommodation (e.g. reimbursement of travel expenses) and delivery by Syrian helpers. Training and structural supervision of peer-refugees was perceived feasible and acceptable. These findings are further supported by helpers' adherence to the protocol and low participant drop-out from the intervention. Two adverse events unlikely attributable to the trial or intervention were reported by participants, suggesting that PM+ is a safe intervention. However, some participants may have experienced shame from trial participation.

Although the study was not powered to detect significant differences, depression and anxiety symptoms improved in the PM+/CAU group relative to the CAU group, with moderate effect sizes. The study also indicated moderate to large improvements in overall psychosocial functioning, PTSD symptoms and self-identified problems. No significant difference in health service utilisation or costs was observed between groups, but overall costs were significantly higher in PM+/CAU due to PM+ implementation costs. Mean intervention costs ultimately are likely to be lower if trainers and helpers can be retained and continue to deliver PM+ to more refugees over a longer time period. Nonetheless, our exploratory economic analysis suggests PM+ has the potential to be cost-effective from a health system perspective.

The moderate improvements across a broad range of symptoms are in line with previous PM+ trials (Rahman *et al*., 2016; Bryant *et al*., 2017), and support the intervention's transdiagnostic feature (Dawson *et al*., 2015). One key finding is that, although PM+ is not a trauma-focused intervention, it improved PTSD symptomatology in this war-affected sample. This adds to existing literature indicating PTSD symptoms can be successfully treated with brief, non-trauma-focused interventions (Nidich *et al*., 2018; Turrini *et al*., 2019).

This is the first study exploring how PM+ can be delivered by peer-refugees in a high-income country. Although refugees are typically exposed to ongoing post-migration stressors, our study showed that effects were retained up to 3 months. A key strength in this study is the mixed-methods design that enabled us to examine both quantitative outcomes in a rigorous RCT, as well as perceptions of various stakeholders about the acceptability and feasibility of PM+. Another strength is the use of the secondary outcome measure PSYCHLOPS, which examines participant-generated problems, instead of ‘Western’ mental health constructs. Furthermore, we added the WHODAS measure of overall psychosocial functioning to look beyond mental health and psychosocial problems, something often not included in the evaluation of psychosocial interventions for refugees (Turrini *et al*., 2019).

Our study also has a number of limitations. First, we failed to interview study drop-outs in our qualitative evaluation, limiting our insights on barriers to trial participation. Second, although treatment effects and cost-effectiveness results are promising, they should be interpreted with caution as no power calculations were carried out. Furthermore, participants were recruited from a foundation established for Syrian families with resident status living in an urban area, and results might be different for those still awaiting completion of their asylum procedure.

A major practical implication of the present pilot RCT is that the study and PM+ procedures can be successfully carried out among Syrian refugees. The observed low drop-out is promising for a definite RCT. Use of Audio-Computer-Assisted Self-Interview software (e.g. Morina *et al*., 2017) that does not require administration by an assessor may improve blinding.

Longer term follow-up is needed to better assess whether there is an impact on health service utilization and social functioning outcomes such as participation in work and study. With a larger sample size, it will also be possible to estimate changes in quality of life outcomes, using a generic outcome measure such as the Quality Adjusted Life Year (QALY), a metric that has resonance with policy makers. Replication in a fully-powered RCT is needed (see De Graaff *et al*., 2020). Other trials on PM+ with different modes of delivery (e.g. group) will be conducted in the larger STRENGTHS project (Sijbrandij *et al*., 2017). This will strengthen the external validity of trial findings and provide a potential model for scaling up in more and less well-resourced contexts.

## Conclusion

This study indicates that the trial procedures and PM+ delivered by peer-refugee, non-specialist helpers are acceptable, feasible and safe. PM+ is likely effective in improving mental health outcomes and psychosocial functioning in Syrian refugees, and potentially cost-effective. A fully-powered, definitve RCT with longer follow-up is needed.

## Data Availability

The Vrije Universiteit Amsterdam (VU) will keep a central data repository of all data collected in the STRENGTHS project. The data will be available upon reasonable request to the STRENGTHS consortium. Data access might not be granted to third parties when this would interfere with relevant data protection and legislation in the countries participating in this project and any applicable EU legislation regarding data protection. Interested researchers can contact Dr Marit Sijbrandij at e.m.sijbrandij@vu.nl to initiate the process.

## Trial registration

https://www.trialregister.nl/trial/6665.

## Appendices

Appendix 1: Checklists

**Checklist A1.** CONSORT 2010 Checklist of information to include when reporting a pilot or feasibility trial

Section/topic	Item No	Checklist item	Reported on page No
Title and abstract			
	1a	Identification as a pilot or feasibility randomised trial in the title	1
	1b	Structured summary of pilot trial design, methods, results, and conclusions (for specific guidance see CONSORT abstract extension for pilot trials)	1
Introduction			
Background and objectives	2a	Scientific background and explanation of rationale for future definitive trial, and reasons for randomised pilot trial	1–2
	2b	Specific objectives or research questions for pilot trial	2
Methods			
Trial design	3a	Description of pilot trial design (such as parallel, factorial) including allocation ratio	2
	3b	Important changes to methods after pilot trial commencement (such as eligibility criteria), with reasons	N/A
Participants	4a	Eligibility criteria for participants	2
	4b	Settings and locations where the data were collected	2
	4c	How participants were identified and consented	2
Interventions	5	The interventions for each group with sufficient details to allow replication, including how and when they were actually administered	2–3
Outcomes	6a	Completely defined prespecified assessments or measurements to address each pilot trial objective specified in 2b, including how and when they were assessed	3
	6b	Any changes to pilot trial assessments or measurements after the pilot trial commenced, with reasons	4
	6c	If applicable, prespecified criteria used to judge whether, or how, to proceed with future definitive trial	N/A
Sample size	7a	Rationale for numbers in the pilot trial	2
	7b	When applicable, explanation of any interim analyses and stopping guidelines	N/A
Randomisation:			
Sequence generation	8a	Method used to generate the random allocation sequence	2
	8b	Type of randomisation(s); details of any restriction (such as blocking and block size)	2
Allocation concealment mechanism	9	Mechanism used to implement the random allocation sequence (such as sequentially numbered containers), describing any steps taken to conceal the sequence until interventions were assigned	2
Implementation	10	Who generated the random allocation sequence, who enrolled participants, and who assigned participants to interventions	2
Blinding	11a	If done, who was blinded after assignment to interventions (for example, participants, care providers, those assessing outcomes) and how	2
	11b	If relevant, description of the similarity of interventions	N/A
Statistical methods	12	Methods used to address each pilot trial objective whether qualitative or quantitative	3
Results			
Participant flow (a diagram is strongly recommended)	13a	For each group, the numbers of participants who were approached and/or assessed for eligibility, randomly assigned, received intended treatment, and were assessed for each objective	3–4 + Fig. 1
	13b	For each group, losses and exclusions after randomisation, together with reasons	3–4 + Fig. 1
Recruitment	14a	Dates defining the periods of recruitment and follow-up	3–4
	14b	Why the pilot trial ended or was stopped	4
Baseline data	15	A table showing baseline demographic and clinical characteristics for each group	5(Table 1)
Numbers analysed	16	For each objective, number of participants (denominator) included in each analysis. If relevant, these numbers should be by randomised group	3–8 + Fig. 1
Outcomes and estimation	17	For each objective, results including expressions of uncertainty (such as 95% confidence interval) for any estimates. If relevant, these results should be by randomised group	5–8
Ancillary analyses	18	Results of any other analyses performed that could be used to inform the future definitive trial	5–8
Harms	19	All important harms or unintended effects in each group (for specific guidance see CONSORT for harms)	5
	19a	If relevant, other important unintended consequences	N/A
Discussion			
Limitations	20	Pilot trial limitations, addressing sources of potential bias and remaining uncertainty about feasibility	8
Generalisability	21	Generalisability (applicability) of pilot trial methods and findings to future definitive trial and other studies	8–9
Interpretation	22	Interpretation consistent with pilot trial objectives and findings, balancing potential benefits and harms, and considering other relevant evidence	8–9
	22a	Implications for progression from pilot to future definitive trial, including any proposed amendments	8–9
Other information			
Registration	23	Registration number for pilot trial and name of trial registry	9
Protocol	24	Where the pilot trial protocol can be accessed, if available	N/A
Funding	25	Sources of funding and other support (such as supply of drugs), role of funders	9
	26	Ethical approval or approval by research review committee, confirmed with reference number	2

**Checklist A2.** Consolidated Health Economic Evaluation Reporting Standards (CHEERS) Checklist

Section/topic	#	Recommendation	Reported on page #/line #
TITLE AND ABSTRACT			
Title	1	Identify the study as an economic evaluation or use more specific terms such as ‘cost-effectiveness analysis’, and describe the interventions compared	1
Abstract	2	Provide a structured summary of objectives, perspective, setting, methods (including study design and inputs), results (including base case and uncertainty analyses), and conclusions	1
INTRODUCTION			
Background and objectives	3	Provide an explicit statement of the broader context for the study Present the study question and its relevance for health policy or practice decisions	1–2
METHODS			
Target population and subgroups	4	Describe characteristics of the base case population and subgroups analysed, including why they were chosen	2
Setting and location	5	State relevant aspects of the system(s) in which the decision(s) need(s) to be made	2
Study perspective	6	Describe the perspective of the study and relate this to the costs being evaluated	3
Comparators	7	Describe the interventions or strategies being compared and state why they were chosen	2–3
Time horizon	8	State the time horizon(s) over which costs and consequences are being evaluated and say why appropriate	3
Discount rate	9	Report the choice of discount rate(s) used for costs and outcomes and say why appropriate	3
Choice of health outcomes	10	Describe what outcomes were used as the measure(s) of benefit in the evaluation and their relevance for the type of analysis performed	3
Measurement of effectiveness	11a	*Single study-based estimates*: Describe fully the design features of the single effectiveness study and why the single study was a sufficient source of clinical effectiveness data	2–3
	11b	*Synthesis-based estimates*: Describe fully the methods used for identification of included studies and synthesis of clinical effectiveness data	N/A
Measurement and valuation of preference based outcomes	12	If applicable, describe the population and methods used to elicit preferences for outcomes	N/A
Estimating resources and costs	13a	*Single study-based economic evaluation*: Describe approaches used to estimate resource use associated with the alternative interventions. Describe primary or secondary research methods for valuing each resource item in terms of its unit cost. Describe any adjustments made to approximate to opportunity costs	2–4
	13b	*Model-based economic evaluation*: Describe approaches and data sources used to estimate resource use associated with model health states. Describe primary or secondary research methods for valuing each resource item in terms of its unit cost. Describe any adjustments made to approximate to opportunity costs	N/A
Currency, price date, and conversion	14	Report the dates of the estimated resource quantities and unit costs. Describe methods for adjusting estimated unit costs to the year of reported costs if necessary. Describe methods for converting costs into a common currency base and the exchange rate	3
Choice of model	15	Describe and give reasons for the specific type of decision-analytical model used. Providing a figure to show model structure is strongly recommended	N/A
Assumptions	16	Describe all structural or other assumptions underpinning the decision-analytical model	N/A
Analytical models	17	Describe all analytical methods supporting the evaluation. This could include methods for dealing with skewed, missing, or censored data; extrapolation methods; methods for pooling data; approaches to validate or make adjustments (such as half cycle corrections) to a model; and methods for handling population heterogeneity and uncertainty	N/A
RESULTS			
Study parameters	18	Report the values, ranges, references, and, if used, probability distributions for all parameters/Report reasons or sources for distributions used to represent uncertainty where appropriate. Providing a table to show the input values is strongly recommended	Appendix
Incremental costs and outcomes	19	For each intervention, report mean values for the main categories of estimated costs and outcomes of interest, as well as mean differences between the comparator groups. If applicable, report incremental cost-effectiveness ratios	Table 3 and 4
Characterising uncertainty	20a	*Single study-based economic evaluation*: Describe the effects of sampling uncertainty for the estimated incremental cost and incremental effectiveness parameters, together with the impact of methodological assumptions (such as discount rate, study perspective)	8+ Appendix
	20b	*Model-based economic evaluation*: Describe the effects on the results of uncertainty for all input parameters, and uncertainty related to the structure of the model and assumptions	N/A
Characterising heterogeneity	21	If applicable, report differences in costs, outcomes, or cost-effectiveness that can be explained by variations between subgroups of patients with different baseline characteristics or other observed variability in effects that are not reducible by more information	N/A
DISCUSSION			
Study findings, limitations, generalisability, and current knowledge	22	Summarise key study findings and describe how they support the conclusions reached. Discuss limitations and the generalisability of the findings and how the findings fit with current knowledge	8–9
Other			
Source of funding	23	Describe how the study was funded and the role of the funder in the identification, design, conduct and reporting of the analysis. Describe other non-monetary sources of support	9
Conflicts of interest	24	Describe any potential for conflict of interest of study contributors in accordance with journal policy. In the absence of a journal policy, we recommend authors comply with International Committee of Medical Journal Editors recommendations	9

**Checklist A3.** Individual PM+ helper's component checklist

SESSION 1					
No.	Item	Components Checklist	COMPLETED?		NOTES
			YES	NO	
1.1	Conduct **Introductions** and explain **Confidentiality**	1.1a – Introduce yourself			
		1.1b – Explain concept of confidentiality, including information about when confidentiality can be broken			
		1.1c – Answer participant questions about PM+ intervention and sessions if needed			
		1.1d – Ask permission for making audio recordings of the sessions (sign IC form)			
1.2	Introduce **What is PM+?**	1.2a – Explain PM+ intervention to the participant			
		1.2b – Discuss participant's reasons for and challenges to attending PM+ sessions			
		1.2c – Support participant to manage any obstacles to attending sessions			
1.3	Conduct **What is Adversity?**	1.3a – Define adversity using participant's examples			
		1.3b – Discuss and normalize common reactions to adversity			
		1.3c – Discuss how PM+ aims to help participants manage their problems			
1.4	Teach and practice **Managing Stress Exercise**	1.4a – Provide information on how stress affects the body			
		1.4b – Relate the information to participant's physical/tension problems			
		1.4c – Teach and practice breathing from the diaphragm/stomach (show balloon)			
		1.4d – Practice slow breathing together			
		1.4e – Discuss challenges and difficulties			
1.5	Use appropriate **psychosocial communication skills**	1.5a – Appropriate eye contact, facial expression, and body			
		1.5b – Demonstrate a non-judgmental attitude			
		1.5c – Appropriate use of non-verbal communication			
		1.5d – Communicate concern and validate participant			
1.6	Incorporate **safety management skills**	1.6a – Review for suicidality if necessary			
		1.6b – Identify potentials risks of harm to self or others			
		1.6c – Use techniques for acute management of risk and provide referral			
1.7	**Closing Procedures**	1.7a – Review session and schedule home practice			
		1.7b – Information of next session (remind date, time, place and strategy)			
**SESSION 2**					
**No.**	**Item**	**Components Checklist**	**COMPLETED?**		**NOTES**
			**YES**	**NO**	
2.1	Incorporate **safety management skills**	2.1a – Review for suicidality if necessary			
		2.1b – Identify potentials risks of harm to self or others			
		2.1c – Use techniques for acute management of risk and provide referral			
2.2	Welcome and review **Managing Stress**	2.2a – Welcome the participant back			
		2.2b – Discuss questions participant has about Session 1			
		2.2c – Review participant's Managing stress home practice			
		2.2d – Help managing any difficulties with home practice			
2.3	Introduce **Managing Problems**	2.3a – Introduce strategy Managing problems for practical problems			
		2.3b – Explain each of the 7 steps			
		2.3c – Help the participant to apply the strategy to a chosen problem			
		2.3d – Help the participant develop and action plan			
		2.3e – Give participant Managing Problems handout			
2.4	Practice **Managing Stress**	2.4a – Practice slow breathing together			
		2.4b – Discuss challenges and difficulties			
2.5	Uses appropriate **psychosocial communication skills**	2.5a – Appropriate eye contact, facial expression, and body			
		2.5b – Demonstrate a non-judgmental attitude			
		2.5c – Appropriate use of non-verbal communication			
		2.5d – Communicate concern and validate participant			
2.6	Incorporate **safety management skills**	2.6a – Review for suicidality if necessary			
		2.6b – Identify potentials risks of harm to self or others			
		2.6c – Use techniques for acute management of risk and provide referral			
2.7	**Closing Procedures**	1.7a – Review session and schedule home practice			
		1.7b – Information of next session (remind date, time, place and strategy)			
**SESSION 3**					
**No.**	**Item**	**Components Checklist**	**COMPLETED?**		**NOTES**
			**YES**	**NO**	
3.1	Incorporate **safety management skills**	3.1a – Review for suicidality if necessary			
		3.1b – Identify potentials risks of harm to self or others			
		3.1c – Use techniques for acute management of risk and provide referral			
3.2	Welcome and review **Managing Stress**	3.2a – Welcome the participant back			
		3.2b – Discuss questions participant has about previous sessions			
		3.2c – Review participant's Managing stress home practice			
		3.2d – Help manage any difficulties with home practice			
3.3	Review **Managing Problems**	3.3a – Discuss participant's experiences of completing their Action Plan for Managing Problems			
		3.3b – Respond to and manage any difficulties (e.g. unable to complete, encountered problems when completing)			
		3.3c – Help participant apply strategy to continue managing the same problem or a new problem			
3.4	Introduce **Get Going and Keep Doing** and the Inactivity cycle	3.4a – Introduce Get Going and Keep Doing strategy			
		3.4b – Show the Inactivity Cycle and explain			
		3.4c – Discuss how inactivity cycle can be broken			
		3.4d – Give participant Get Going and Keep Doing handout			
3.5	Apply **Get Going Keep Doing** with an enjoyable activity	3.5a – Help participant to select an **enjoyable** activity			
		3.5b – Help participant break down their activity into small steps			
		3.5c – Help participant develop their action plans			
3.6	Practice **Managing Stress**	3.6a – Practice slow breathing together			
		3.6b – Discuss challenges and difficulties			
3.7	Use appropriate **psychosocial communication skills**	3.7a – Appropriate eye contact, facial expression, and body			
		3.7b – Demonstrate a non-judgmental attitude			
		3.7c – Appropriate use of non-verbal communication			
		3.7d – Communicate concern and validate participant			
3.8	Incorporate **safety management skills**	3.8a – Review for suicidality if necessary			
		3.8b – Identify potentials risks of harm to self or others			
		3.8c – Use techniques for acute management of risk and provide referral			
3.9	**Closing Procedures**	3.9a – Review session and schedule home practice			
		3.9b – Information of next session (remind date, time, place and strategy)			
**SESSION 4**					
**No.**	**Item**	**Components Checklist**	**COMPLETED?**		**NOTES**
			**YES**	**NO**	
4.1	Incorporate **safety management skills**	4.1a – Review for suicidality if necessary			
		4.1b – Identify potentials risks of harm to self or others			
		4.1c – Use techniques for acute management of risk and provide referral			
4.2	Welcome and review **Managing Stress**	4.2a – Welcome the participant back			
		4.2b – Discuss questions participant has about previous sessions			
		4.2c – Review participant's Managing stress home practice			
		4.2d – Help manage any difficulties with home practice			
4.3	Review **Managing Problems**	4.3a – Discuss participant's experiences of completing their Action Plan for Managing Problems			
		4.3b – Respond to and manage any difficulties (e.g. unable to complete, encountered problems when completing)			
		4.3c – Help participant apply strategy to continue managing the same problem or a new problem			
4.4	Review **Get Going Keep Doing**	4.4a – Discuss participant's experiences of completing their Action Plan for Get Going Keep Doing			
		4.3b – Respond to and manage any difficulties (e.g. unable to complete, encountered problems when completing)			
		4.3c – Help participant apply strategy to continue with the same activity or start a new activity (task-oriented activity)			
4.5	Introduce **Strengthening Social Support**	4.5a – Define Strengthening Social Support			
		4.5b – Discuss different social supports and how they can be helpful			
		4.5c – Give participant Strengthening Social Supports handout			
4.6	Apply **Strengthening Social Support**	4.6a – Help participant identify how they can strengthen their social support			
		4.6b – Help participants develop an action plan			
		4.6c – Rehearse with participants what they will do/say in their action plan if applicable			
4.7	Practice **Managing Stress**	4.7a – Practice slow breathing together			
		4.7b – Discuss challenges and difficulties			
4.8	Use appropriate **psychosocial communication skills**	4.8a – Appropriate eye contact, facial expression, and body			
		4.8b – Demonstrate a non-judgmental attitude			
		4.8c – Appropriate use of non-verbal communication			
		4.8d – Communicate concern and validate participant			
4.9	Incorporate **safety management skills**	4.9a – Review for suicidality if necessary			
		4.9b – Identify potentials risks of harm to self or others			
		4.9c – Use techniques for acute management of risk and provide referral			
4.10	**Closing Procedures**	3.10a – Review session and schedule home practice			
		3.10b – Information of next session (remind date, time, place and strategy)			
**SESSION 5**					
**No.**	**Item**	**Components Checklist**	**COMPLETED?**		**NOTES**
			**YES**	**NO**	
5.1	Incorporate **safety management skills**	5.1a – Review for suicidality if necessary			
		5.1b – Identify potentials risks of harm to self or others			
		5.1c – Use techniques for acute management of risk and provide referral			
5.2	Welcome and review **all PM+ home practice**	5.2a – Welcome the participant back			
		5.2b – Discuss questions participant has about previous sessions			
		5.2c – Review Managing Stress home practice			
		5.2d – Review Managing Problems home practice			
		5.2e – Review Get Going Keep Doing home practice			
		5.2f – Review Strengthening Social Support home practice			
		5.2g – Manage any difficulties participants had with any of their home practice			
5.3	Complete **Staying Well**	5.3a – Congratulate the participant on finishing PM+			
		5.3b – Discuss participant improvements and areas for continued work			
		5.3c – Emphasize importance of continuing to practice PM+ strategies, using learning a new language example			
		5.3d – Discuss potential future problems and how participant can respond			
5.4	Complete **How to help others**	5.4a – Use the case examples to discuss how the participant could help others experiencing problems			
		5.4b – Discuss their responses and correct any misunderstandings			
5.5	Complete **Looking Forward**	5.5a – Review goals that were not achieved			
		5.5b – Discuss how the participant can work towards these goals			
		5.5c – Help participants to identify new goals			
5.6	Use appropriate **psychosocial communication skills**	5.6a – Appropriate eye contact, facial expression, and body			
		5.6b – Demonstrate a non-judgmental attitude			
		5.6c – Appropriate use of non-verbal communication			
		5.6d – Communicate concern and validate participant			
5.7	Incorporate **safety management skills**	5.7a – Review for suicidality if necessary			
		5.7b – Identify potentials risks of harm to self or others			
		5.7c – Use techniques for acute management of risk and provide referral			
5.8	**Closing Procedures**	5.8a – Review session and schedule home practice			
		5.8b – Information of next session (remind date, time, place and strategy)			

**Appendix 2: Tables Table A1.** Unit cost (2018 euros)

Type of cost	Unit cost	Unit	Source
Community health worker (assumed to be equivalent to nurse practitioner)	€18	Per consultation	Hakkaart-van Roijen *et al*. (2015)
Community-based doctor	€34	Per consultation	Hakkaart-van Roijen *et al*. (2015)
Psychiatrist	€98	Per consultation	Hakkaart-van Roijen *et al*. (2015)
Psychologist	€67	Per consultation	Hakkaart-van Roijen *et al*. (2015)
Psychiatric nurse	€18	Per consultation	Hakkaart-van Roijen *et al*. (2015)
Social worker (maatschappelijk werk)	€68	Per consultation	Hakkaart-van Roijen *et al*. (2015)
Physiotherapist	€34	Per session	Hakkaart-van Roijen *et al*. (2015)
Other health professionals (assumed same as community health worker)	€18	Per consultation	Hakkaart-van Roijen *et al*. (2015)
Hospital inpatient stays – general health (weighted average for general and university hospitals)	€495	Per day	Hakkaart-van Roijen *et al*. (2015)
Hospital outpatient services – general health (weighted average for general and university hospitals)	€95	Per visit	Hakkaart-van Roijen *et al*. (2015)
Hospital A&E	€269	Per visit	Hakkaart-van Roijen *et al*. (2015)
Medications (Tramadol)	€0.11	Per 50 mg capsule	Zorginstituut Nederland (2020)
Medications (Duloxetine)	€0.06	Per 30 mg capsule	Zorginstituut Nederland (2020)
Minimum wage rate aged 20 (July 2018)	€51.51	Per day	Government of the Netherlands (2020)
Minimum wage rate aged 21 (July 2018)	€63.39	Per day	Government of the Netherlands (2020)
Minimum wage rate aged 22+ (July 2018)	€74.58	Per day	Government of the Netherlands (2020)

**Table A2.** Qualitative analysis themes and related quotes

Themes	Interviewee	#	Selected quotes
Theme 1 Experiences with the PM+ intervention			
- Positive effects	P1	Q1.1	The positive point is that they [helpers] can understand the problem. And they can find solutions. They can think in a different way. A way different from ours, different from the routine way of thinking. They provide relaxation.
	P3	Q1.2	First thing is the breathing, because as I get angry, this breathing technique is very useful.
	P6	Q1.3	Like language, I can attend free courses or [visit] the neighbour's house. (…) It was one of the [practical] problems I can solve. But the problem that they may remove me back to Syria, no one can predict what would happen after a year and half. So it does not have a solution.
	P3	Q1.4	In the past my husband was telling me ‘let's go somewhere’ and I was saying ‘no I don't feel like.’ So I am breaking this routine and maybe I am resting and maybe enjoying or maybe the opposite but the positives are more than the negatives.
	P4	Q1.5	From the inside I felt better.
	KI3	Q1.6	They [PM+ participants] gave examples like; that they used the daily [PM+] strategies and that they carried out the breathing exercises on a daily basis. And they also mentioned examples of how they benefitted from it. That was a good check for me; what has it [PM+] brought?
	KI4	Q1.7	And they [helpers] kept repeating that it [PM+] was really useful for themselves. When they started doing the [PM+] training. For example, understanding their own fears and experiences but also in their own families.
- Adherence to strategies	H2	Q1.8	With some [participants] it [PM+ strategies] went smooth, with others you had to repeat it and practice it again and make it smaller, make it softer, so that they would at some point taste and feel the experience.
	P1	Q1.9	I almost continued for one month after the end of the [intervention]. I kept using the same style. But after, I didn't continue. I forgot about it to be honest. But sometimes I think about it. When I have problems (…) Then, I remember the breathing technique.
- Duration of PM +	P4	Q1.10	Yes, I wanted us to be in contact and he keeps asking me about my problems and follows me up.
	H5	Q1.11	For one participant who does not have much stress it is doable, but for the other, it might be too brief.
	H3	Q1.12	And I think that some people need nothing more than the intervention itself to continue with the strategies, but maybe for others it would be good to match them to something that will help them and reminds them of [PM+]. I don't know how, maybe via trainings or a YouTube channel or a website or something, yes.
- Barriers/facilitators - Time/emotional burden - Accommodation - Stigma	KI1	Q1.13	We [SNTR] tried to take away all the logistical barriers [to participate in PM+] like covering their transport to the location and making it like a free programme.
	H1	Q1.14	But what's good is that we always heard that if it is too much you can tell this and you don't have to take any new participants for a while or you can say that you can only have one participant per week.
	P1	Q1.15	The appointment [of PM+ sessions] was exact and everything was perfect.
	P6	Q1.16	I told two people [about my participation in the project] I felt that they started laughing (…) I felt that they are making fun of it. So, I didn't tell anyone after.
Theme 2 Views on the helper			
- Rapport and trust	P3	Q2.1	In Syria, people with mental problems are called crazy. The [helper] isn't a psychiatrist but someone you speak to about your problems and maybe she can help you finding a solution. This is how I liked the program and liked participating.
	P2	Q2.2	I considered her a friend. Not a durable friendship but friendship in the session's time. I was feeling comfortable talking to her.
- Similar culture/experiences/language	P1	Q2.3	Because he [helper] is from the same country I was able to communicate with him easily. And speak my native language Arabic without any language barriers.
	KI3	Q2.4	And especially the group of people that is not familiar in talking about their mental health, I think it will be so hard for them to find the right words to express themselves in their second language.
	P6	Q2.5	When I talk about anything, he [helper] could directly understand why I am talking like this. Maybe he went through same conditions like me.
- Helper is ‘neutral’	H5	Q2.6	In PM+ they will be with Syrian people, from their own culture, from their own language, but not people that they know, not family or so, so they will talk.
	KI5	Q2.7	And some [participants] don't tell their families [about PM+]. They come here in secret; they don't want anyone to know.
	KI2	Q2.8	We have two teams [at SNTR]: a South team and a North team. Some [PM+] helpers were working in the South team and others in the North. And if it was possible, I would exchange them; if a participant was from team South, I would try to find a helper from the North.
	P3	Q2.9	I felt good with her [helper] and safe…because maybe I can't share these things that are inside me with someone that I know.
- Competence	P2	Q2.10	Her [helper] answers were convincing. You feel that she is a competent helper.
	P1	Q2.11	Even if he is not professional, he was well trained.
	P5	Q2.12	But as I told you, I couldn't take him [helper] as seriously as I would with a professor, as it is coming from a person not specialized and hasn't studied at the university.
Theme 3 Experiences of training and supervision			
- Experiences of PM + training and supervision	KI5	Q3.1	But it is, as trainer, really, yes, hard work. Because you have to learn a lot [as helper] and you notice that during supervision afterwards they still grow. You have to repeat the basics for quite some time. Because they [PM+ helpers] might start doing other things.
	H1	Q3.2	Of course the first and or second time were exciting. We often had to turn to the protocol, so to say. Up to now we do that, but I notice that there are many things we say automatically, without asking help we do it ourselves. You can learn a lot about the PM+ strategies from practical experience. But the first knowledge by a specialist and having enough time and enough explanation through the training was very good.
- Supervision as: - Further training ground - Space to discuss cases - Source of emotional support	H4	Q3.3	Of course, we also had weekly supervision, so we talk about it. We are very clear and open about the difficulties we face, how we can better approach it, from how other colleagues dealt with it.
	H2	Q3.4	And with the supervisor of course you can always ask about it, learn more, ask questions about how you can deal with it in another way.
	H4	Q3.5	But some people maybe they need extra help, but we also indicate this if we notice this. So we talk about it with our supervisor and then we will see what is best for our participants.
	KI5	Q3.6	Yes because they [helpers] also recognize the stories [of PM+ participants]. So that was something we took into consideration. Yes. So on the one hand it was good in terms of recognition, but on the other hand it was difficult for them [helpers].
	H5	Q3.7	[The PM+ participants] sometimes come with very heavy stories and we are people, too, of course. But I always try to protect myself and find distraction, or in supervision it is good to talk about it of course. I also talk with other colleagues, that helps me too. To express my story.

H, helper; KI, key informant; P, PM+ participant; SNTR, Stichting Nieuw Thuis Rotterdam.

Theme 3: Experiences of training and supervision

The PM+ supervisor described the weekly supervisions as ‘hard work’ because the helpers had no background in psychology (KI5). Both supervisor and helpers experienced that further practice of PM+ skills during supervision increased helpers' ability to model the strategies to participants and adhere to the protocol (Q3.1–2). Helpers described supervision as a way ‘to learn from each other’ (H2) and ‘improve their approach’ (H4) (Q3.3). Communication between supervisors and helpers was in Dutch, but some of the role-play was in Arabic (to practice how PM+ will work with participants).

Individual case monitoring was mainly used to support tailoring PM+ to the individual (Q3.4), and to detect and potentially refer those with possibly more severe mental health problems (e.g. substance abuse) to professional care (Q3.5). Several KIs, however, expressed concerns about the current waiting lists for such professional help.

Recognition of participants' stories was helpful though potentially burdensome for helpers (Q3.6). They explained that hearing about other people's problems could be challenging, but that supervision and talking to colleagues helped them (Q3.7). The supervisor emphasised the importance of self-care during supervision, given the similarity of experiences (emotional burden), and because helpers carried out the sessions alongside their regular work for SNTR (time burden). Both helpers and the supervisor spoke about the importance of knowing ‘your limits’ as helper. One helper was asked to briefly put PM+ as extra activity in her regular work ‘on hold’ due to signs of burn-out.

**Table A3.** Missing data pattern

		Time point		
Group	Outcome measure	Baseline (*N* = 60)	Post-assessment (*N* = 53)	3-month follow-up (*N* = 54)
Whole sample	HSCL-25, *n* of missing items (%)	6 (0.4)	7 (0.5)	2 (0.1)
	WHODAS 2.0, *n* of missing items (%)	1 (0.1)	3 (0.5)	1 (0.2)
	PCL-5, *n* of missing items (%)	9 (0.8)	0 (0)	0 (0)
	PSYCHLOPS, *n* of missing items (%)	7 (2.9)	1 (0.5)	1 (0.5)
	**Total, *n* of missing items (%)**	**23 (0.6)**	**11 (0.3)**	**4 (0.1)**
PM+/CAU	HSCL-25, *n* of missing items (%)	4 (1.1)	4 (0.6)	1 (0.1)
	WHODAS 2.0, *n* of missing items (%)	0 (0)	1 (0.3)	1 (0.3)
	PCL-5, *n* of missing items (%)	7 (1.2)	0 (0)	0 (0)
	PSYCHLOPS, *n* of missing items (%)	7 (5.8)	0 (0)	0 (0)
	**Total, *n* of missing items (%)**	**18 (0.9)**	**5 (0.3)**	**2 (0.1)**
CAU	HSCL-25, *n* of missing items (%)	2 (0.6)	3 (0.5)	1 (0.2)
	WHODAS 2.0, *n* of missing items (%)	1 (0.3)	2 (0.6)	0 (0)
	PCL-5, *n* of missing items (%)	2 (0.3)	0 (0)	0 (0)
	PSYCHLOPS, *n* of missing items (%)	0 (0)	1 (1.0)	1 (1.0)
	**Total, *n* of missing items (%)**	**5 (0.3)**	**6 (0.4)**	**2 (0.1)**

PM/CAU, Problem Management Plus and care as usual group; CAU, care as usual control group.

The percentage of *missed assessments* (unit non-response; Brick and Kalton, 1996) in the study was 7.2%. Eighty-seven per cent (86.7%) of participants completed all three assessments. There were more assessment skips in the CAU group (13% at both post and 3-month follow-up) than in the PM+/CAU group (10% at post and 6.7% at 3-month follow-up). Reported reasons for non-attendance across assessments were ‘prefer to withdraw’ (*n* = 3), ‘lack of time’ (*n* = 3), ‘abroad/unavailable’ (*n* = 1), and ‘no approval from spouse’ (*n* = 1).

The percentage of *missing items* (item non-response; Brick and Kalton, 1996) across the primary outcome variable HSCL-25 and secondary outcome variables WHODAS 2.0, PCL-5 and PSYCHLOPS varied between 0.1–2.9% (baseline), 0–0.5% (post-assessment) and 0–0.5% (3-month follow-up). We assumed data to be missing at random (Little and Rubin, 2002). Table A3 presents missing values per outcome variable.

We used pro-rated, single imputations for outcome measures with missing items. We did not impute unit non-response, as linear mixed-models in R handle missing outcome data (Raudenbush, 2001).

**Table A4.** Summary statistics and results from mixed-model analysis of primary and secondary outcomes without PM+ non-completers

		Descriptive statistics, M (s.d.)		Mixed model analysis^a^		
Outcomes	Time point	PM+/CAU (*n* = 28)	CAU (*n* = 30)	Difference in LS mean (95% CI)	*p*-value	Effect size^a^
HSCL-25 total	Baseline	2.43 (0.56)	2.55 (0.65)			
	Post-assessment	1.86 (0.58)	2.38 (0.65)	0.45 (0.287–0.611)	0.007	0.62
	Follow-up	1.92 (0.63)	2.42 (0.59)	0.48 (0.317–0.651)	0.005	0.59
HSCL anxiety	Baseline	2.34 (0.64)	2.41 (0.63)			
	Post-assessment	1.76 (0.62)	2.22 (0.71)	0.40 (0.229–0.569)	0.021	0.49
	Follow-up	1.92 (0.64)	2.38 (0.64)	0.43 (0.255–0.607)	0.017	0.56
HSCL depression	Baseline	2.48 (0.57)	2.64 (0.72)			
	Post-assessment	1.93 (0.66)	2.48 (0.68)	0.49 (0.311–0.663)	0.006	0.49
	Follow-up	1.92 (0.64)	2.45 (0.63)	0.52 (0.345–0.697)	0.004	0.57
WHODAS 2.0	Baseline	32.25 (7.83)	30.52 (7.51)			
	Post-assessment	24.19 (9.16)	27.77 (9.37)	3.32 (1.10–5.64)	0.141	0.54
	Follow-up	23.45 (6.43)	30.15 (10.05)	6.55 (4.28–8.82)	0.004	0.97
PCL-5	Baseline	35.59 (18.14)	37.25 (17.11)			
	Post-assessment	21.41 (16.06)	34.50 (15.47)	11.18 (6.71–15.65)	0.014	0.59
	Follow-up	20.25 (17.85)	34.12 (17.14)	13.35 (8.85–18.41)	0.006	0.75
PSYCHLOPS	Baseline	15.44 (2.56)	15.47 (3.95)			
	Post-assessment	9.81 (5.92)	13.52 (4.89)	3.28 (1.87–4.69)	0.022	0.61
	Follow-up	9.62 (5.81)	15.12 (7.40)	5.34 (3.51–7.17)	0.005	0.81

HSCL-25 = 25-item Hopkins Symptoms Checklist (range item-mean = 1–4, higher scores indicate elevated anxiety or depression); WHODAS 2.0 = WHO Disability Assessment Schedule 2.0 (range 12–60, higher scores indicate worse functional impairment); PCL-5 = PTSD Checklist for DSM-5 (range 0–80, higher scores indicate greater severity); PSYCHLOPS = Psychological Outcomes Profiles (range 0–20, higher scores indicate poorer outcome); M = mean; s.d. = standard deviation.

a Effect sizes are determined by calculating the difference between the estimated means (corrected for baseline) divided by the raw pooled standard deviation.

**Table A5.** Reliable change index at post-assessment and 3-month follow-up for the HSCL-25 (completers only)

	Post-assessment		Follow-up	
RCI	PM + /CAU (*n* = 27)	CAU (*n* = 26)	PM + /CAU (*n* = 28)	CAU (*n* = 26)
Recovered, *n* (%)^a^	2 (7.4%)	0 (0%)	3 (10.7%)	0 (0%)
Improved without recovery, *n* (%)^b^	11 (40.7%)	7 (26.9%)	11 (39.3%)	6 (23.1%)
Deteriorated, *n* (%)^b^	0 (0%)	1 (3.8%)	0 (0%)	2 (7.7%)
No change, *n* (%)	14 (51.9%)	18 (69.2%)	14 (50.0%)	18 (69.2%)

RCI, reliable change index.

a The Clinical Significant Change cut-off for the HSCL-25 (total scale) was calculated by subtracting 2 s.d. of the baseline M for the full sample.

b The RCI for the HSCL-25 (total score) was calculated using the baseline s.d. for the full sample and baseline Cronbach's *α* as test-retest reliability coefficient (Jacobson and Truax, 1991). Recovered = clinical significant reliable change; Improved without recovery = no clinical significant reliable change; Deteriorated = reliable change with worsening of symptoms; No change = no reliable change.
